# *Tsx* Produces a Long Noncoding RNA and Has General Functions in the Germline, Stem Cells, and Brain

**DOI:** 10.1371/journal.pgen.1002248

**Published:** 2011-09-01

**Authors:** Montserrat C. Anguera, Weiyuan Ma, Danielle Clift, Satoshi Namekawa, Raymond J. Kelleher, Jeannie T. Lee

**Affiliations:** 1 Howard Hughes Medical Institute, Boston, Massachusetts, United States of America; 2 Department of Molecular Biology, Massachusetts General Hospital, Boston, Massachusetts, United States of America; 3 Department of Genetics, Harvard Medical School, Boston, Massachusetts, United States of America; 4 Center for Human Genetic Research, Massachusetts General Hospital, Boston, Massachusetts, United States of America; 5 Department of Neurology, Harvard Medical School, Boston, Massachusetts, United States of America; 6 Division of Reproductive Sciences, Cincinnati Children's Hospital Medical Center, Cincinnati, Ohio, United States of America; Stanford University School of Medicine, United States of America

## Abstract

The *Tsx* gene resides at the X-inactivation center and is thought to encode a protein expressed in testis, but its function has remained mysterious. Given its proximity to noncoding genes that regulate X-inactivation, here we characterize *Tsx* and determine its function in mice. We find that *Tsx* is actually noncoding and the long transcript is expressed robustly in meiotic germ cells, embryonic stem cells, and brain. Targeted deletion of *Tsx* generates viable offspring and X-inactivation is only mildly affected in embryonic stem cells. However, mutant embryonic stem cells are severely growth-retarded, differentiate poorly, and show elevated cell death. Furthermore, male mice have smaller testes resulting from pachytene-specific apoptosis and a maternal-specific effect results in slightly smaller litters. Intriguingly, male mice lacking *Tsx* are less fearful and have measurably enhanced hippocampal short-term memory. Combined, our study indicates that *Tsx* performs general functions in multiple cell types and links the noncoding locus to stem and germ cell development, learning, and behavior in mammals.

## Author Summary

The X-linked gene *Tsx* is located within the X-inactivation center and is thought to encode a protein expressed in testis, yet its function is not known. Here we show that *Tsx* is actually a noncoding RNA, a new member of the large noncoding RNA family expressed from the X. Tsx is abundantly expressed in meiotic germ cells, embryonic stem cells, and brain. Targeted deletion of *Tsx* generates viable offspring, litter ratios are smaller than expected, X-inactivation is mildly affected (in embryonic stem cells), and male animals have smaller testes due to germ cell apoptosis. Mutant embryonic stem cells are severely growth-retarded and differentiate poorly with elevated cell death. Deletion of this noncoding RNA alters mouse behavior, with animals displaying less fear and enhanced short-term memory. Our study indicates that *Tsx* performs general functions in multiple cell types and links the noncoding locus to stem and germ cell development, learning, and behavior in mammals.

## Introduction

**Figure 1:**
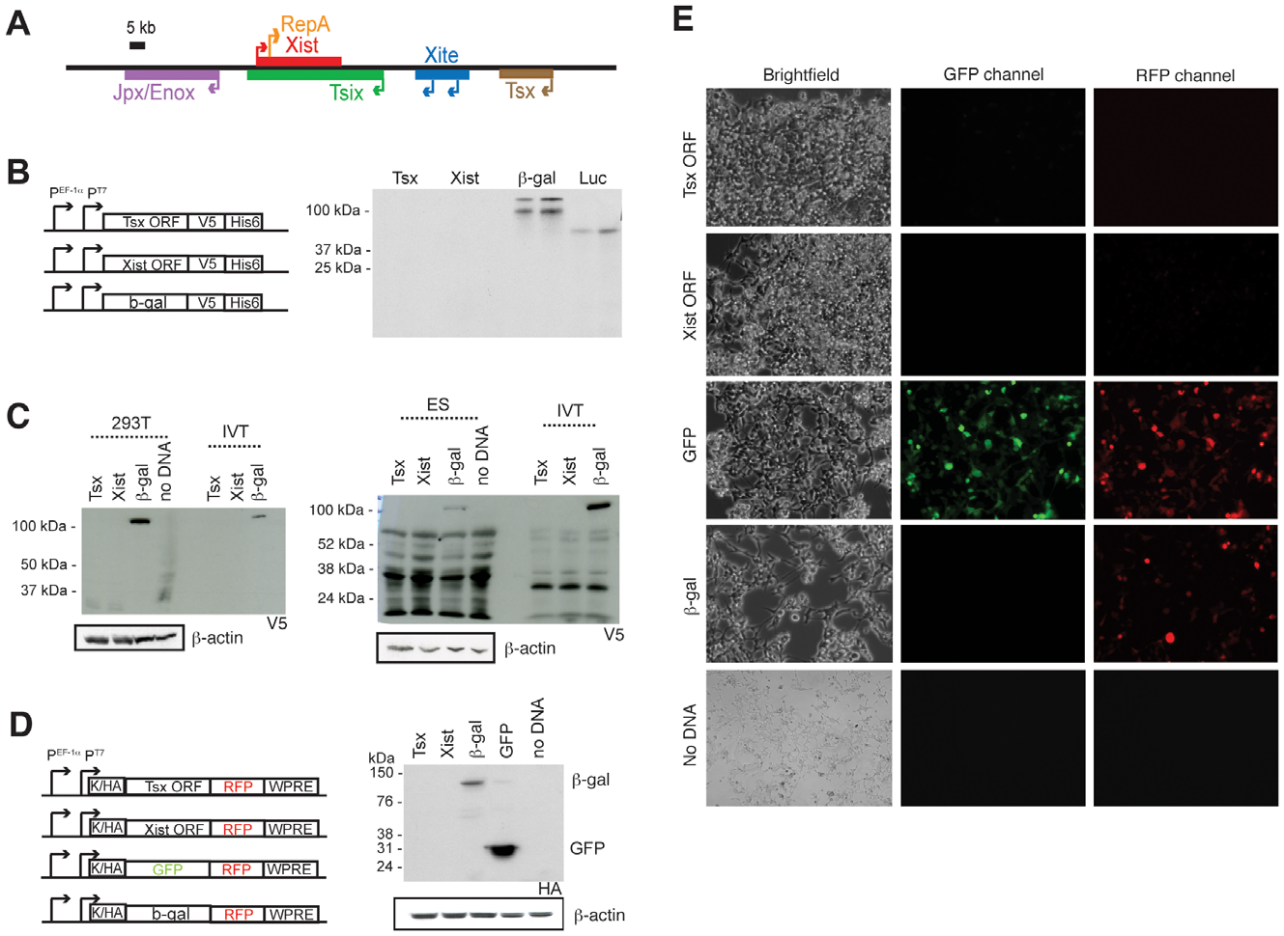
*Tsx* is a long noncoding RNA. (A) Map of the X-inactivation center, with positions and transcriptional orientations of each gene. (B) ORFs from *Tsx* and *Xist*, similar in length and lacking stop codons, were cloned into expression vectors in frame with a V5 and His6 epitope tags under control of the human EF-1a promoter (P^EF-1a^). After programming in rabbit Reticulocyte lysate, the reactions were run on an SDS-page gel. The luciferase control was supplied by the *in vitro* transcription/translation kit. (C) Western blots of human 293T cells and mouse ES cells (J1) after transfection with constructs shown in (B). *In vitro*-translated reactions were run alongside on an SDS-PAGE and then detected using anti-V5 and ß-actin antibodies. ß-actin, loading control. (D) Western blot of 293T cells transfected with constructs shown were detected using anti-HA and ß-actin antibodies. K, Kozack sequence; HA, HA tag. (E) Live-cell imaging after 48 hours of transfection with constructs in (D). Images were taken at 10× magnification and exposure times of 11 msec. The experiment was performed twice with similar results. One corresponding Western blot is shown in (D).

The X-inactivation center (*Xic*) controls X-chromosome inactivation (XCI) and is enriched for genes that produce long noncoding RNAs (ncRNA) (Figure 1A) [1], [2], [3]. Several have been shown to regulate XCI. The 17-kb Xist RNA is induced at the onset of XCI and silences the X-chromosome as the RNA spreads *in cis* along the chromosome. *Xist* expression is controlled both negatively and positively [4], [5], [6], [7], . *Xist*'s antisense partner, *Tsix*, controls X-chromosome counting and allelic choice, blocking *Xist* induction on the future active X [8], [9], [11]. The upstream locus, *Xite*, is required to sustain *Tsix* expression on the future active X during XCI [12], [13]. Two other noncoding RNAs, Jpx and RepA, function as activators and are required for transcriptional induction of Xist RNA [7], [14]. Together, these five noncoding genes span <200 kb of sequence. Several studies have suggested that additional regulators of XCI reside in close proximity to this core domain of the *Xic* [5], [15], [16].

Located immediately upstream of *Xite* is *Tsx* (Testes-specific X-linked), a gene of some interest owing to its unique location and evolutionary history [16], [17], [18]. *Tsx* is absent in marsupials and arose some 150 million years ago in eutherians during the transition from imprinted to random XCI in mammals. Although absent in marsupials, *Tsx* partially aligns to genomic sequences in chickens, specifically within three exons of a coding gene of unknown function called *Fip*1l2 [18]. This finding suggests that vertebrate *Fip*1l2 is the evolutionary precursor of eutherian *Tsx*. Because *Tsx* is only partially conserved among eutherian mammals, it is believed to be a pseudogene in some species such as human, cow, and dog [15]. Nevertheless, human *TSX* shares significant homology to mouse *Tsx*, particularly within exons 1, 3, 4, 5, and 6 [15]. Several Tsx splice variants have been reported in the mouse, including a predominant species of 794-nt transcript with a 432-nt ORF predicted to encode a highly acidic protein of 156 amino acids [17]. Two ORF-less species of 351 and 540 nt have also been described. Curiously, immunostaining with anti-sera raised against Tsx protein suggested exclusive expression in pre-meiotic germ cells in pubertal mouse testes, though Tsx mRNA is not detected until meiosis [17]. In adult animals, Tsx mRNA is predominantly observed in testis [16], specifically in Sertoli cells [17]. Tsx RNA is not detectable in the female germline, but can be seen in 2-cell mouse embryos and throughout preimplantation development [19].

These previous analyses therefore suggest a male germline protein originating from the *Xic*, whose expression coincides with or precedes the meiotic period. With respect to XCI, meiotic mechanisms in the male have been proposed to guide imprinting of the paternal X-chromosome in daughter embryos and to thereby direct paternal-specific silencing of X in the early embryo – a form of XCI known as “imprinted XCI” [20], [21], [22], [23], [24]. Here we examined the idea that *Tsx* may function during imprinted XCI. Contrary to expectation, generation of a *Tsx* mouse knockout showed no effects on imprinting. However, in the course of analysis, we learned that *Tsx* is actually noncoding and uncover several functions for *Tsx* in mouse development and behavior.

## Results

### *Tsx* is noncoding

Together with *Tsx*'s location within the RNA-enriched *Xic*, several observations led us to revisit the question of whether *Tsx* is a coding gene. First, *Tsx*'s genic ancestry mirrors that of the well-established Xist RNA, which is also proposed to have evolved by pseudogenization of a coding gene (*Lnx*3) [18]. Second, between mouse and rat, the *Tsx* cDNAs are 79% identical, yet the 5′ UTR unexpectedly display significantly greater homology (89% identity) [16], [17], indicating conservation of noncoding elements within the gene. Third, *Tsx* lacks a Kozak sequence for translation initiation. Finally, immunostaining with an antiserum against a Tsx peptide produced a tissue staining pattern inconsistent with its RNA profile [17]. Taken together, these findings suggest that Tsx RNA may not be translated. Indeed, Tsx protein has never been isolated from cells, and our continuous search for Tsx peptides has not identified matches in the extensive mouse or human proteomic databases to date (http://world-2dpage.expasy.org/repository/; http://reprod.njmu.edu.cn/cgi-bin/2d/2d.cgi).

To investigate the protein-coding potential of *Tsx*, we cloned the putative *Tsx* open reading frame (ORF) (432-nt sequence generating a predicted protein of ∼20 kDa) in frame with a C-terminal V5 tag in a mammalian expression vector and tested whether it could be translated in a cell-free translation system (Figure 1B). A peptide corresponding to the predicted protein was never observed. Similar results were observed for a hypothetical ORF within the established noncoding RNA, Xist (ORF of 468 nt located 2 kb downstream of *RepA*). By contrast, ß-galactosidase (ß-gal) and luciferase proteins were consistently produced. These results indicated that the *Tsx* ORF is not translated *in vitro*.

To determine whether the *Tsx* ORF could be translated *in vivo*, we transiently transfected human 293T cells and mouse ES cells and then performed Western blot analyses using anti-V5 antibodies. Again, no proteins of predicted size (∼20 kDa) were detected for *Tsx* and *Xist* in either cell type, whereas ß-gal was readily detected (Figure 1C). To exclude the possibility that the C-terminal V5 tag was masked and therefore undetectable by Western analysis, we cloned the *Tsx* and *Xist* ORFs (lacking stop codons) into an expression vector containing an N-terminal HA tag and a C-terminal red fluorescence protein (RFP) fusion for live-cell detection of potential protein products (Figure 1D). To increase transcript stability and enhance translation, we also cloned in a 3′ WPRE and a 5′ Kozak sequence, respectively. [Note: Both the *Tsx* and *Xist* lack Kozak sequences [16]]. Following transient transfection into 293T cells, RFP was detected from neither the *Tsx*- nor *Xist*-transfections (Figure 1E). By contrast, RFP was easily detected from positive controls in which RFP is fused to either GFP or ß-gal. Western blot analysis using an anti-HA antibody confirmed expression of ß-gal∶RFP and GFP∶RFP fusions, but not Tsx∶RFP or Xist∶RFP. We conclude that *Tsx* does not produce a protein and its transcription produces a long noncoding RNA.

### *Tsx* expression patterns

**Figure 2:**
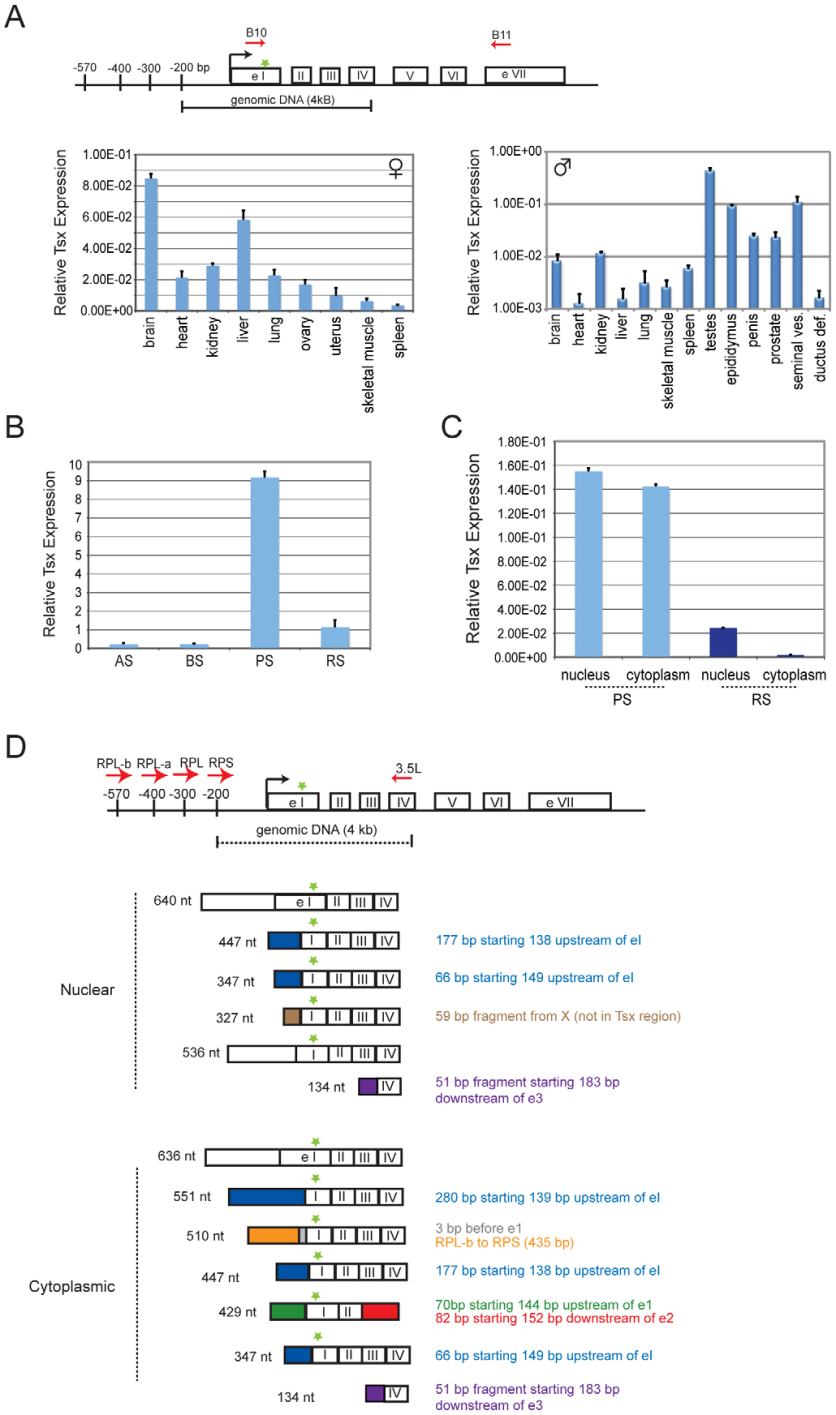
Tsx expression patterns. (A) Map of *Tsx* with locations of primer pairs used in PCR analysis. ATG start site is indicated by a green star. Quantitative RT-PCR analysis of *Tsx* RNA in adult female and male tissues (age matched littermates). Standard deviations refer to triplicate measures for each tissue sample from at least two different animals. Tsx primers B10 and B11 (spanning exons 1 through 7) were used for amplification. (B) Quantitative RT-PCR analysis of *Tsx* RNA during spermatogenesis. Type A Spermatogonia (AS), Type B Spermatogonia (BS), Pachytene Spermatocytes (PS), Round Spermatids (RS). The same primer set from (A) was used for amplification, and similar results were observed with primers Tsx 1 and Tsx 2 (also spanning exons 1 and 7). (C) Quantitative PCR analysis of *Tsx* RNA for nuclear and cytoplasmic fractions of pachytene spermatocytes and round spermatids. The same primer sets from (B) were used to amplify Tsx RNA (shown are results using Tsx B10 and Tsx B11). (D) Map indicating alternative splicing at the 5′ end of the transcripts cloned from PS. There is no evidence for alternative splicing between exons 2–7. Therefore, exons 5–7 are not shown for the splice variants. Tsx primers denoted by red arrows, and the ATG start site is indicated by a green star. Alternative transcripts were amplified using the same reverse primer (Tsx 3.5L; exon 4) and four different forward primers (RPL-b, RPL-a, RPL, RPS) located upstream of exon 1.

We next examined *Tsx* expression patterns *in vivo*. Quantitative RT-PCR (qRT-PCR) analysis showed that *Tsx* is widely expressed in adult tissues (Figure 2A). In female mice, highest levels were found in the brain. In male mice, the RNA was expressed at 8-fold lower levels in brain relative to that in female brain. Furthermore, whereas the female gonadal levels were unremarkably low, male gonadal expression was 10- to 100-times higher than in the male brain. High-level expression was generally observed for tissues of the male reproductive tract (e.g., epididymus, prostate). The testes nonetheless exhibited greatest expression, consistent with previous reports [17].

We then isolated male germ cells from the testes and fractionated them by meiotic stage (Figure 2B). qRT-PCR showed that the premeiotic cell types, Type A and B spermatogonial cells (AS, BS), displayed relatively low-level expression. Expression increased 40-fold during meiosis, specifically in pachytene-stage spermatocytes (PS), suggesting *de novo* transcriptional induction of Tsx RNA during this stage. Tsx expression then decreased 9-fold in round spermatids (RS), the earliest post-meiotic stage cells, but steady state levels remained elevated relative to AS and BS stages. RNA at this stage could represent either continued but decreased expression of Tsx RNA or retention of RNA synthesized during the pachytene stage. The massive upregulation of *Tsx* during pachytene is intriguing, given that the sex chromosomes are inactivated during this stage in a process known as ‘meiotic sex chromosome inactivation’ (MSCI) [25]. A previous report of X-linked expression during spermatogenesis revealed that only a handful of genes escaped transcriptional silencing during MSCI [24]. Significantly, *Tsx* is one of the loci that escapes.

To determine its subcellular localization pattern, we separated pachytene spermatocytes and round spermatids into nuclear and cytoplasmic fractions. qRT-PCR demonstrated that Tsx RNA could be found in both compartments during pachytene, but it appears to be predominantly nuclear in round spermatids (Figure 2C). Previous analysis showed the Tsx RNA can occur in 794-, 540-, and 351-nt isoforms [17]. To determine which splice variant occurs in each compartment, we cloned and sequenced cDNAs from the nucleus and cytoplasm, and identified more variants than previously observed, with the variations occurring in exon 1 and upstream exons (Figure 2D; downstream exons appear similar; The Tsx alternative transcript (Tsx RPS) originally described as being tissue-specific [17] appeared to be expressed in all cell types, including in male germ cells. We conclude that Tsx RNA exists as multiple RNA species in pachytene spermatocytes.

### Generation of *Tsx*-null mice

**Figure 3:**
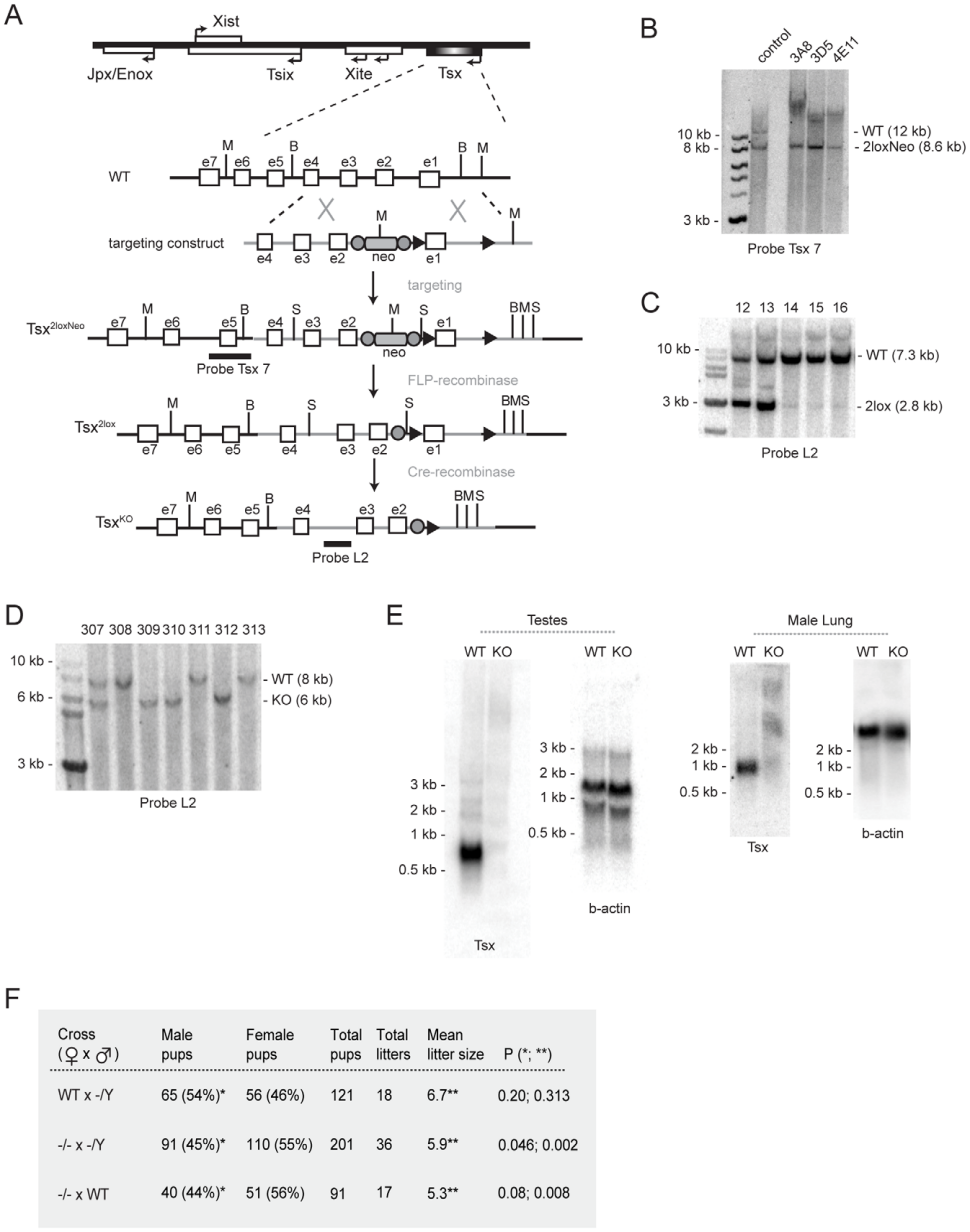
Generation of *Tsx^KO^* mice. (A) Exon-intron structure of the *Tsx* locus (orientation relative to *Xist*) and the targeting strategy. The targeting vector DNA is represented as the grey line and the endogenous DNA is shown as the black line. Restriction enzymes are designated as (M) for *Msc*I, (B) for *BstZ*17I, (S) for *Sph*I. FRT sites, denoted as filled grey circles, flank the PGK-Neomycin selection marker (neo), and loxP sites are denoted as black triangles. Probes used for Southern blotting analyses are denoted as black horizontal bars. (B) Southern blotting of genomic DNA from three positive clones digested with *Msc*I and probed with Tsx probe 7. ‘Control’ corresponds to a sample containing DNA from both wildtype cells (wt) and correctly targeted mutant cells (mutant). Clone 3A8 was selected for blastocyst injection. (C) Southern blotting of genomic tail DNA from pups from mating mutant Tsx^2loxNeo^ male and female mice with FLP-Recombinase expressing animals. DNA was digested with *Sph*I and probed using Tsx probe L2. (D) Southern blotting of genomic tail DNA from mutant animals, generated by mating mutant Tsx^2loxNeo^ animals with EIIA-Cre mice. DNA was digested with *BstZ*17I and probed using Tsx probe L2. (E) Northern blot analyses of male testes and lung tissues from wildtype and mutant littermates. The blots were first probed for *Tsx* then stripped and re-probed for ß-actin. (F) Matings of Tsx-null animals. P-values were calculated using one-tailed Student's t-test assuming equal variance.

To investigate *Tsx* function, we generated *Tsx* knockout mice (*Tsx^KO^*) by homologous targeting in ES cells, blastocyst injection, and production of chimeric mice for germline transmission. The *Tsx^KO^* allele deletes a 2 kb region encompassing exon 1 and the upstream regulatory region (Figure 3A). Following electroporation into male ES cells, three homologously targeted clones were isolated (Figure 3B) and clone 3A8 was used for blastocyst injection. Two chimeric males were obtained and both gave germline transmission of the *Tsx^2loxNeo^* allele. We mated *Tsx^2loxNeo^* offspring to FLP-expressing mice to remove the Neomycin selection marker (Figure 3C) and then crossed the resulting *Tsx^2lox^* offspring to Cre-expressing mice to generate *Tsx^KO^* (Figure 3D). We verified loss of all Tsx RNA isoforms by Northern blot analysis using full-length cDNA probes (Figure 3E) and also by RT-PCR analysis using exon-specific primers across all exonic sequences (data not shown).

### Effects of *Tsx^KO^* in female and male mice

Intercrosses between *Tsx^KO^* mutant mice (−/Y, −/+) and outcrosses to wildtype C57BL/6J generally produced offspring at expected Mendelian ratios and normal mean litter sizes of ∼7 (Figure 3F and data not shown). All offspring were viable and fertile. However, when −/− females were crossed to −/Y males, we observed a mild but statistically significant sex-ratio distortion favoring females and a lower mean litter size (Figure 3F). Similar findings were observed when −/− females were outcrossed to wildtype males. Intercrossing −/Y males to wildtype females did not reproduce these effects. These results suggested that −/− females have mildly reduced fertility and that female births are slightly favored.

**Figure 4:**
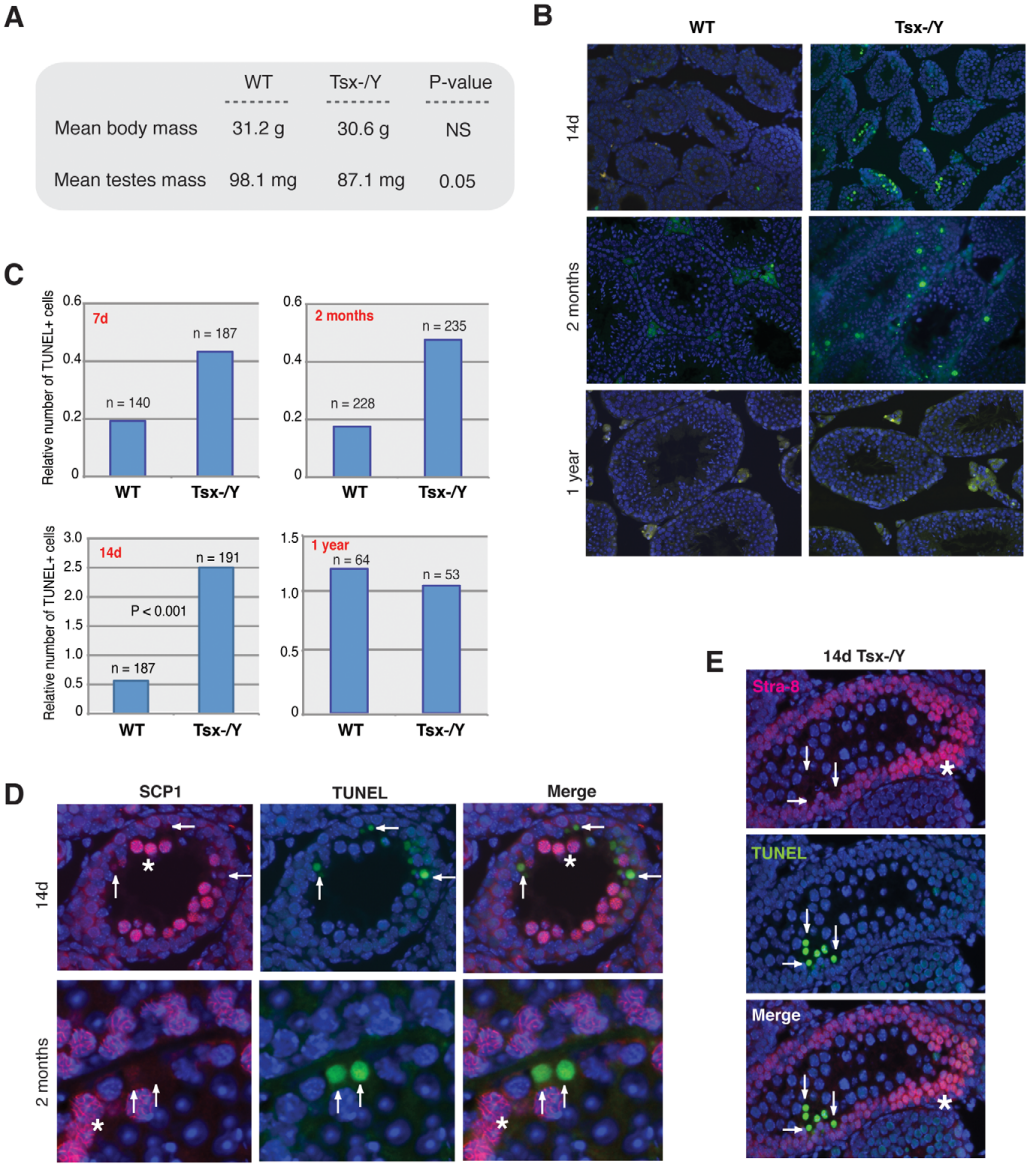
*Tsx^KO^* affects testes size and induces pachytene-specific apoptosis. (A) Average testes and body masses from 8 wildtype and 7 Tsx−/Y littermate animals aged 6 months. P-values were calculated using a one-tailed Student's t-test assuming equal variance. NS (Not Significant). (B) TUNEL staining (green) of seminiferous tubule sections from wildtype and Tsx−/Y littermates aged 7d, 14d, 2 months, and 1 year. Testes were fixed, paraffin embedded, then sectioned. TUNEL-positive cells are shown in green and nuclei stained with DAPI (blue). Three sets of littermates were analyzed yielding similar results; images from one set of littermates are shown. (C) Quantification of the number of TUNEL-positive cells from seminiferous tubule sections. The ratio of TUNEL-positive cells/tubules was determined by counting the number of TUNEL-positive cells within tubules for each field and dividing by the number of tubules per field. The number of tubules (n) counted for each sample is shown. P-values were calculated using Student's t-test. (D) Co-staining for TUNEL (green) and SCP-1 (red) in seminiferous tubule sections from Tsx KO animals aged 14d and 2 months. White stars indicate SCP-1 positive PS, and white arrows denote SCP-1 positive cells. Images were taken at 20×. (E) Co-staining for TUNEL (green) and Stra-8 (red) in tubule sections from Tsx−/Y animals during first wave of meiosis (males aged 14d). White stars indicate Stra-8 positive pre-meiotic cells, and arrows denote TUNEL positive cells that are also Stra-8 negative (meiotic germ cells). Images were taken at 20×.

The fertility of −/Y males was somewhat surprising, given high-level Tsx expression in wildtype pachytene spermatocytes. We therefore asked if there were measurable consequences of deleting *Tsx* in the male germline. We isolated testes from littermate animals (6 months) and found that testes from mutant males were smaller than wildtype littermates (Figure 4A). Histological examination of testicular sections revealed no gross abnormalities at 7d, 14d, 2 month, and 1 year (data not shown). To test whether the smaller testis size was due to loss of germ cells, we performed TUNEL staining of paraffin-embedded testicular sections and found elevated apoptosis between mutant and wildtype animals at 7 days, 14 days, and 2 months (Figure 4B, 4C). The difference was greatest at 14d, coinciding with the first wave of pachytene (meiotic prophase I) during post-natal male development [26]. At this timepoint, there were five times as many apoptotic germ cells in the −/Y testes. Significant differences were also observed as early as 7d at a time when male germ cells prepare to enter the first wave of meiosis, and also at 2 months when male animals reach adulthood. At 1 year, the difference became insignificant.

These results suggested loss of germ cells as the animals first proceed through meiosis. To determine which cell type and what stage of meiosis were affected, we combined TUNEL staining and immunofluorescence for SCP1 (synaptonemal complex protein 1), a combination that enabled us to distinguish between meiotic and non-meiotic cells and also between meiotic cells of different stages. Tubule sections from 14d mutants revealed that TUNEL-positive cells were located 2 to 3 cell layers in from the basal lamina membrane (most mature germ cells are found in the center of a tubule cross-section) and all were SCP1-negative. The TUNEL-positive cells resided in the same cell layer as pachytene spermatocytes and were always positioned next to pachytene spermatocytes that showed robust staining with SCP1 (Figure 4D). Co-staining with Stra-8 [27] showed no overlap between Stra-8-positive and TUNEL-positive cells in 14d tubule sections, thus ruling out pre-meiotic germ cells (Figure 4E). These findings argued that the apoptotic cells were in fact pachytene spermatocytes whose chromatin had become too fragmented for SCP1 staining. Examination of tubules from 2-month old mutant mice showed similar staining patterns (data not shown). To determine if abnormal MSCI could be the cause of germ cell loss, we carried out immunofluorescence with antibodies against H3-K9me3, HP1-γ, γ-H2AX, and HP1β – chromatin modifications that decorate the XY body during MSCI or post-meiotic sex chromatin (PMSC) in round spermatids [24]. No obvious differences between wildtype and mutant cells could be seen (data not shown). We conclude that a subset of pachytene-stage cells inappropriately undergoes apoptosis when *Tsx* is deleted, but the cause of sub-viability is not clear at present.

### Effects of *Tsx^KO^* in ES cells

**Figure 5:**
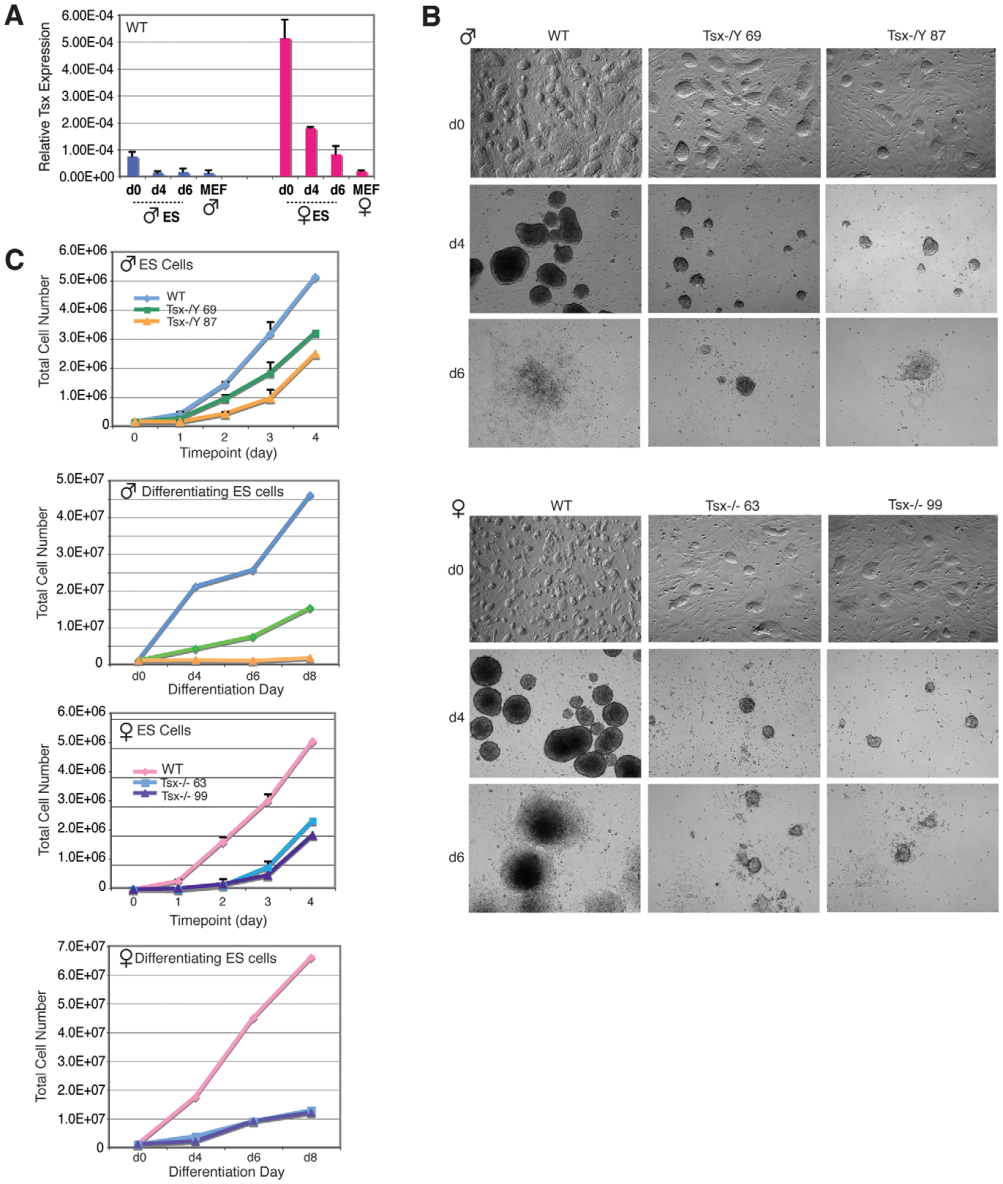
*Tsx* KO ES cells exhibit differentiation defects and cell death. (A) Quantitative RT-PCR analysis of *Tsx* RNA in male (J1) and female (EL16.7) mouse ES cells during differentiation. Mouse embryonic fibroblasts (MEFs). (B) Phase contrast images (all taken at 4×) of male and female Tsx-null undifferentiated ES cells (d0) and EBs at differentiation d4 and d6. ES cells were differentiated by suspension culture using medium lacking LIF in three independent experiments yielding similar results (images from one experiment are shown). (C) Cell growth of undifferentiated and differentiating male and female null ES cell lines. Equal numbers of cells were plated in triplicate on equal numbers of MEFs, then harvested daily and counted using an automated cell counter.

Because *Tsx* is expressed at the 2- and 4-cell stages, and also in blastocyst embryos [19], we examined whether it is also expressed in wildtype ES cells. qRT-PCR revealed that *Tsx* is expressed in both undifferentiated male and female ES cells and that it is downregulated during differentiation (Figure 5A). Female ES cells consistently demonstrated 5- to 10-times more expression than male ES cells. Given *Tsx*'s expression in ES cells, we next asked whether deleting *Tsx* affects ES cell growth and differentiation. We derived *Tsx*−/Y and −/− ES cells from blastocysts resulting from −/−×*Tsx*−/Y matings and obtained two independent female clones and four independent male clones. The growth rates of undifferentiated *Tsx*−/Y and −/− ES cells were significantly affected when compared to wildtype cells for multiple independent clones tested (Figure 5C). Similar growth retardation was observed for both mutant male and female clones when they were differentiated into embryoid bodies (EBs). In general, mutant EBs were much smaller during suspension culture (d0–d4). They also attached poorly to plates during the adherent growth phase (d4–d8) and showed sparse outgrowth compared to wildtype controls (Figure 5B). Although increased cell death was observed in some clones by quantitative assays based on cytotoxicity measurements, cell death could not have been the sole cause of poor growth, as only some male and female mutant clones demonstrated significantly higher cell death (data not shown).

### Effects of *Tsx^KO^* on XCI

**Figure 6:**
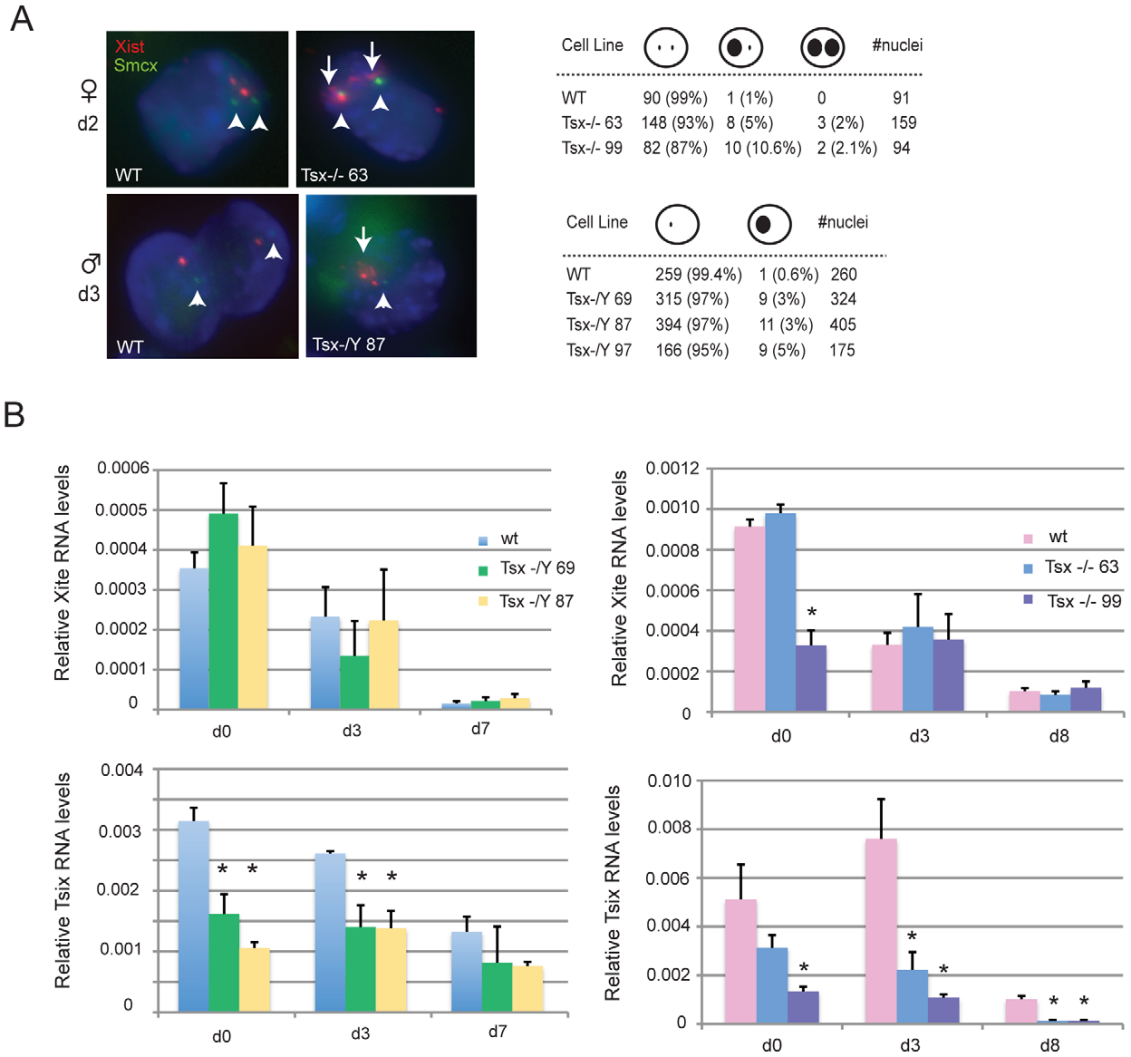
Analysis of Xist, Tsix, and Xite expression in *Tsx^KO^* ES cells. (A) RNA/DNA FISH analysis for *Xist* expression in female and male cells during early differentiation. First, an *Xist* probe was used to detect *Xist* RNA (red) followed by DNA FISH using a Smcx BAC probe (green) to denote the X-chromosome. Arrows denote the presence of *Xist* clouds and the arrowhead denotes the X-chromosome DNA signal from Smcx. All nuclei counted had either 2 green Smcx pinpoints (females) or 1 green pinpoint (males), indicating a diploid nucleus. (B) qRT-PCR analyses of *Tsix*, and *Xite* expression in undifferentiated and differentiating female and male *Tsx^KO^* ES cells. P-values were calculated using one-tailed Student's t-test assuming unequal variance.

Because *Tsx* resides at the *Xic* and is immediately proximal to *Xite* and *Tsix*, we asked if aberrant XCI may be a cause of the ES cell anomalies. Standard assays for X-inactivation choice did not reveal any skewing of allelic choice (data not shown). However, combined RNA/DNA fluorescent in situ hybridization (FISH) showed that Xist RNA was aberrantly upregulated in a small fraction of cells during cell differentiation (Figure 6A). This was observed in both male and female ES cells and in multiple independent clones. Whereas wildtype male cells almost never upregulate Xist RNA, *Tsx*-deficient male cells showed Xist clusters in 3–5% of differentiating cells on d3. Similarly, whereas wildtype female cells usually only showed one Xist RNA cloud during differentiation, mutant female cells displayed two RNA foci in 5–10% on d2. To avoid counting tetraploid cells (which would have 4 X-chromosomes and appropriately show two Xist RNA clouds), Xist RNA FISH was combined with an *Smcx* BAC DNA FISH to determine X-chromosome number (Figure 6A). Only diploid cells were scored in this assay. It should be noted that in both male and female mutants, the ectopic *Xist* cluster was relatively loose and generally not as large as those typically seen on the inactive X. We conclude that deleting *Tsx* partially affects *Xist* regulation in ES cells.

Given the ectopic Xist expression and because *Xist* is known to be regulated by *Xite* and *Tsix*, we next asked if *Tsx*'s effect on *Xist* may occur through *Tsix and Xite* by examining expression of Tsix and Xite RNAs in two independent male and female *Tsx^KO^* ES cell lines. Interestingly, whereas the knockout had little to no significant effect on *Xite* expression, it caused a significant downregulation of *Tsix* expression at all timepoints and in both male and female cells (Figure 6B). These results suggest that a *Tsx* knockout may induce ectopic *Xist* expression by blunting the upregulation of *Tsix* during ES cell differentiation. Thus, *Tsx* may be a positive regulator of *Tsix*.

### Deleting *Tsx* affects male behavior and learning

**Figure 7:**
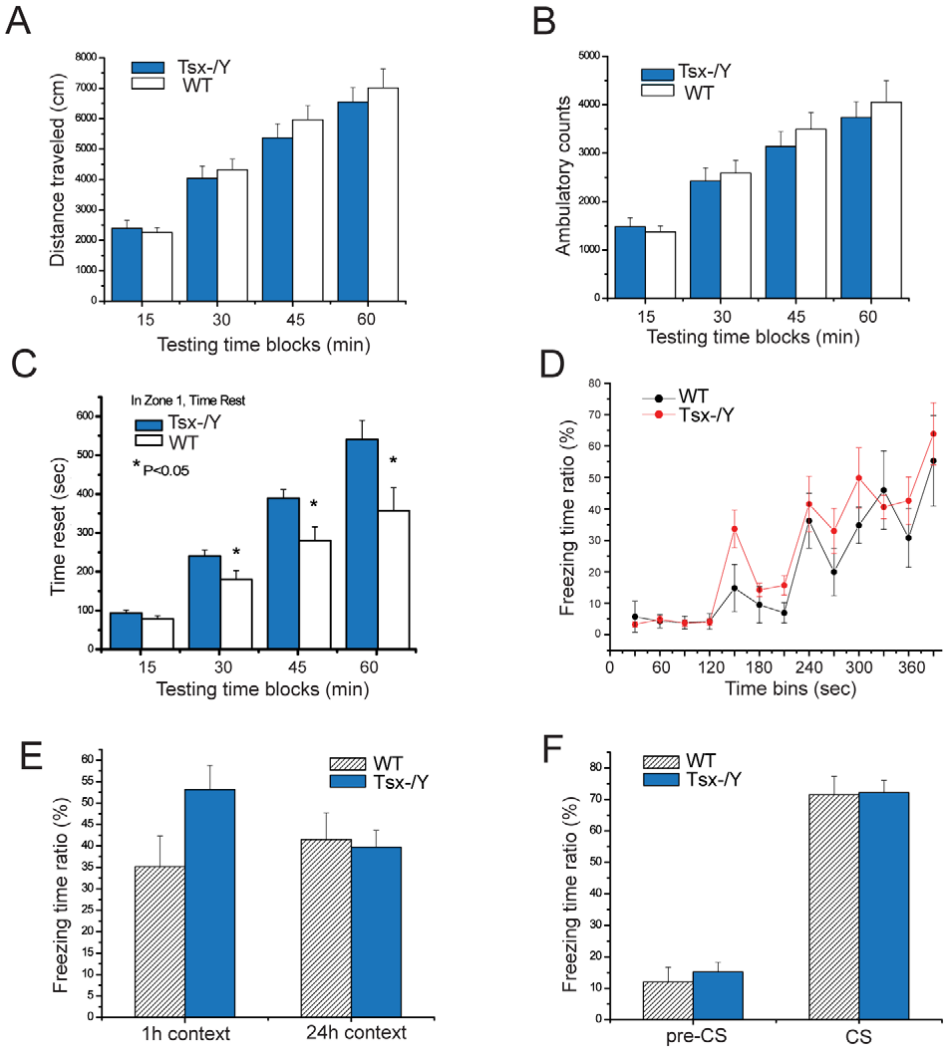
Tsx deletion affects behavior and short-term hippocampal-dependent memory consolidation in male mice. (A) Open field test for the total distance traveled for wildtype (n = 9) and Tsx −/Y (n = 9) animals. (B) Open field test for ambulatory counts for the same group of male animals. (C) Open field test quantifying the amount of time spent in the center (Zone 1) of the chamber. * indicates P<0.05 for one-way ANOVA tests. (D) Conditioning response curve for fear conditioning tests for wildtype (n = 5) and Tsx −/Y (n = 7). (E) Contextual fear conditioning tests for 1 h and 24 h after training. The mean percentage of time spent freezing for two independent experiments is shown, for both wildtype (n = 10) and Tsx −/Y (n = 12) male animals. (F) Cued fear conditioning tests. The same groups of animals were tested in novel environmental chambers 48 hr after training (for two independent experiments). The mean percentage of time spent freezing is shown for animals before tone presentation (“pre-CS”) and during the phasic presentation of the tone (“CS”).

The observation that *Tsx* expression in somatic tissues was highest in brain suggested that *Tsx* may regulate neural processes underlying behavior. Knockout animals displayed no gross neuromuscular defects or disturbances in gait. As a first step toward assessing a possible role for *Tsx* in cognitive function, we therefore subjected *Tsx* knockout mice and littermate controls to a series of well-established behavioral paradigms. Exploratory behavior was first analyzed in the open field task, which is sensitive to changes in locomotor activity, stereotypy and anxiety [28]. *Tsx* knockout and age-matched wildtype littermates displayed similar values for total distance and ambulatory counts, indicating that *Tsx* deletion does not affect locomotor activity (Figure 7A, 7B). Mice typically explore the periphery of the chamber and avoid the center. Although *Tsx* knockout mice spent significantly more time at rest in the chamber's central zone (P<0.05; Figure 7C), they displayed a normal overall preference for the peripheral relative to the central zone in terms of total occupancy (Figure S1A). To investigate further the possibility of altered anxiety levels, we examined the behavior of *Tsx* knockout and wildtype littermates in the elevated plus maze task [29], [30]. The total occupancy and number of entries in the open and closed arms of the maze were similar between knockout and wildtype animals (Figure S1B, S1C), arguing against any generalized changes in anxiety-related behavior as a result of *Tsx* deficiency.

To explore a possible role for *Tsx* in cognitive function, we next analyzed associative learning and memory for conditioned fear. In contextual fear conditioning, mice learn to associate a particular experimental chamber or “context” with a mild foot shock following a single brief training session, such that subsequent exposure to the context elicits a fear response measured by immobility or “freezing”. In cued fear conditioning, mice learn to associate an experimental tone with a mild foot shock, thereafter displaying a fear response when presented with the tone in a novel context. Contextual fear memory requires both the hippocampus and amygdala, whereas cued fear memory is amygdala-dependent but hippocampus-independent [31], [32]. Assessment of fear responses at retention intervals of 1 hour and 24 hours after training distinguishes short-term memory and long-term memory for conditioned fear [33], [34]. Age-matched male *Tsx* knockout and wildype littermates were subjected to contextual fear conditioning then tested for the memory of the experimental context at 1 hour and 24 hours following the conditioning. Prior to conditioning, knockout and wildtype animals exhibited similarly low baseline levels of freezing, supporting the conclusion from the open field task that *Tsx* deletion does not affect locomotor activity (Figure 7D). Both groups of mice also displayed similar freezing responses to presentation of the tone and foot shocks during the training sessions. Interestingly, knockout mice exhibited significantly higher levels of freezing when tested at a retention interval of 1 hour (Figure 7E; wildtype 35%, mutant 53%, p = 0.059). This difference was not observed at a retention interval of 24 hours, indicating that knockout mice display a selective enhancement of short-term contextual memory. No differences were detected between knockout and wildtype mice for cued fear conditioning (Figure 7F).

Behavioral tests are typically carried out in male mice because estrus cycles in female mice are difficult to synchronize and are known to affect testing. However, because Tsx levels were higher in brain tissue of female mice, we made an effort to test female knockouts. In attempts to control for estrus cycling, female animals were group-housed (4 animals per cage) for 2 weeks prior to testing (note: co-habitation partially synchronizes estrus cycles; we could not synchronize pharmacologically because exogenous hormones have profound effects on behavior). We found no differences between wildtype and *Tsx*−/− female mice in either the open field test or the fear conditioning tests (Figure S2 and data not shown). There are two potential explanations for this result. First, *Tsx* may indeed have no effect on female behavior. Second, effects of deleting *Tsx* may have been masked by fluctuating estrus cycles. From the collective evidence, we conclude that *Tsx* deletion likely affects specific enhancement of short-term hippocampal memory, at least in male mice.

## Discussion

Here we have described a novel long ncRNA expressed from the *Tsx* locus, a gene that was previously thought to encode a protein. Tsx RNA is expressed at varying levels from many tissues. Multiple isoforms of the RNA can be observed, with the predominant species being the one encoded by all seven exons, regardless of cell type. The greatest amount and isoform diversity are seen in pachytene spermatocytes, a cell type in which the X-chromosome from which Tsx is expressed is otherwise transcriptionally inactive. Indeed, Tsx is only one of four genes and several microRNAs that escape transcriptional silencing during MSCI in the male germline [24], [35]. Surprisingly, knocking out *Tsx* neither affected MSCI nor male fertility. It also did not affect imprinted XCI. However, *Tsx* mutants showed a number of reproductive defects, including pachytene-specific apoptosis, smaller testes size, mildly reduced litter sizes and a mild sex ratio distortion related to maternal *Tsx* deficiency. The subtlety of some of these defects may be attributed to the possibility that Tsx is functionally redundant with regards to its meiotic role during male and female germ cell development.

*Ex vivo*, the mutants exhibit poor stem cell growth and both male and female ES cells display aberrant *Xist* upregulation in a small fraction of cells. We suggest that this may be due to downregulation of *Tsix*, a known repressor of *Xist*. Thus, *Tsx* may be a positive regulator of *Tsix* and consequently an indirect repressor of *Xist*. Further investigation is required to pinpoint the mechanism. Aberrant *Xist* expression may in part explain the poor viability during cell differentiation, but we do not believe that XCI consequences are the sole cause, as the level of ectopic Xist expression is not high. Strangely, in spite of these measurable defects in ES cell growth, knockout male and female embryos are viable, with only a slight sex ratio distortion and reduction in litter size. *Tsx* mutants must be able to compensate *in vivo* for the stem cell-related defects seen *ex vivo*. *In vivo*, the growth of the epiblast lineage in the ICM niche must provide what culture media cannot. The possibility of functional redundancy with other stem cell regulators can also be entertained here.

A most intriguing phenotype to arise from the mutants is the effect on behavior and cognition. We were prompted to examine a possible role for *Tsx* in brain function based on observation that brain exhibits the highest levels of *Tsx* expression among somatic tissues examined. Interestingly, our analysis revealed enhanced short-term memory for contextual fear conditioning in *Tsx* knockout male mice. [No effects were seen in female mice, but the results may have been confounded by varying estrus cycles in the subjects]. Since assessment of learning and memory in fear conditioning depend on fear responses measured by immobility, it was important to consider any potentially confounding effects of *Tsx* deletion on locomotor activity, anxiety or differential responses to the conditioning regimen. The normal behavior displayed by knockout mice in the open field, elevated plus maze and fear conditioning training sessions argues that *Tsx* deletion causes a specific alteration in hippocampal memory. Some noncoding RNAs in the brain (*e.g.* BC1, BC200) regulate gene expression post-transcriptionally by binding to mRNAs and repressing their translation [36], [37]. While the specific regulatory functions of *Tsx* are presently unknown, it is tempting to speculate that *Tsx* deletion may lead to constitutive derepression of the expression of gene products required for memory acquisition. Further studies will be needed to elucidate the molecular mechanisms by which *Tsx* may regulate learning and memory in the mammalian brain.

## Materials and Methods

### *Tsx* ORF transient transfections and Western blot analysis

The *Tsx* ORF (480 bp; consisting of 48 bp before the ATG start codon to the position before the stop codon) was cloned from a mouse testes cDNA library and amplified using primers *Tsx* cDNA F2 5′-AGCACCCACCTAGACTTGGG-3′ and *Tsx* exon 7 no stop R 5′-ATCAGTTGGGTTCATGGCAC-3′. Two different ORFs within the mouse *Xist* gene were cloned. The *Xist* ORF lacking a stop codon found within exon 1 (469 bp) was amplified using primers 5′-ATGCTCTGTGTCCTCTATCAGA-3′and 5′-GAAGTCAGTATGGAGGGGGT-3′, and the *Xist* ORF (489 bp; also lacking a stop codon) within exon 7 was amplified using primers 5′-ATGTTCTCCTGCATGTTCT-3′ and 5′- GAATACAAGAGAGACACAGA-3′. The *Tsx* and *Xist* exon 1 ORFs were cloned in frame with the V5 epitope of the pEF1/V5-His vectors (Invitrogen). These constructs contain a T7 promoter upstream of the ATG start codon, and 1 µg of each construct was used with the T7 TNT Rabbit Reticulocyte Lysate In-vitro Transcription/Translation kit (Promega), and 1/5 of the reaction mix was run on a 12% SDS-PAGE gel. In addition, the *Tsx* ORF, *Xist* exon 7 ORF, ß-galactosidase cDNA, and GFP cDNA (all lacking stop codons) were cloned in frame with RFP into the pPS-EF1-LCS-T2A vector (System Biosciences). Mouse ES cells and human 293T cells (50% confluent in a 10 cm plate) were transfected with 24 µg of the various DNA constructs using Lipofectamine 2000 according to the manufacter's protocol (Invitrogen), and cells were harvested 48 hours later. The cell pellets were sonicated three times (10 seconds each) in a lysis buffer containing 10 mM Trish pH 7.5, 150 mM NaCl, 5 mM EDTA, 1% Triton X-100, 20 mM 2-mercaptoethanol, and 1 mM PMSF. Protein lysates (25 µg/lane) were run on a 10% or 12% SDS-PAGE gel, then transferred to a PVDF membrane. The membrane was blocked with 5% non-fat dry milk in PBS containing 0.1% Tween-20, then incubated with either a mouse monoclonal (Invitrogen) or rabbit polyclonal (Novus Biologicals) V5 epitope antibody (diluted 1∶5,000) or HA epitope antibody (diluted 1∶10,000) at 4°C overnight. The next day the membrane was washed four times with PBS/Tween, then incubated with anti-rabbit horseradish peroxidase-labeled secondary antibody (diluted 1∶20,000) for 1 hour at room temperature. The membrane was washed again with PBS/Tween, then visualized using the West Pico Chemilluminescence system (Pierce).

### Cellular fractionation and germ cell purification

For the fractionation experiments, cells were resuspended in lysis buffer (10 mM Tris pH 8.4, 1.5 mM MgCl, 140 mM NaCl), then 4 µL of 5% Nonidet P40 (diluted in PBS) was added on ice. Aliquots were taken at 5 min, 10 min, 15 min, and 30 min, and centrifuged for 3 min at 2400 rpm. The supernatant (cytoplasm) was transferred to a new tube and Trizol-LS was added. The pellet (nuclei) was washed 2× with PBS, then Trizol was added. Germ cells from testes of 7d C57/BL/6J (for isolation of Type A and B spermatogonia) and 2 month old CD-1 males (for isolation of pachytene spermatocytes and round spermatids) were isolated using gravity sedimentation with the STA-PUT device as described previously [24], [26]. PS and RS fraction purtity was >95% as judged by phase optics (for qRT-PCR in Figure 2C); RNA for qRT-PCR of Tsx in Figure 2B was also used in Reference 24. RNA from the fractionated cell populations was isolated using Trizol (Invitrogen).

### RT-PCR and real-time PCR analysis

Tissues from 2–4 different wildtype male and female C57BL/6J animals (2 months old) were immediately placed in Trizol after dissection, then homogenized using a Qiagen TissueLyser, and the RNA was cleaned up using the RNeasy Mini kit (Qiagen). All RNA was treated with Turbo DNAse (Ambion) following the ‘rigorous’ protocol. Reverse transcription of RNA to cDNA was performed using SuperScript III (Invitrogen), 1 µg RNA and random hexamers. All cDNA was diluted 1∶5 prior to qPCR, except for cDNA from male and female animal tissues, which was not diluted. For real-time PCR analyses, Tsx cDNA (exons 1–7) was amplified using two primer sets: Tsx B10 and Tsx B11 [17] and Tsx 1 5′-ATTAAGCAGGCAGGCAGAAA-3′; Tsx 2 5′-TGCGGTGATTTTCATTTTGA-3′. For transcript amplification in PS (Figure 2D) we used a reverse primer (Tsx 3.5L; within exon 4) and 4 different forward primers (RPS-F, RPL-F, RPLb-F, RPLa-F; upstream of exon 1). ß-actin primers: 5′- CCGTGAAAAGATGACCCAG-3′ (F) and 5′-TAGCCACGCTCGGTCAGG-3′ (R). Tsx primers: 3.5L 5′-AGCTTGGCAAGTGTCCTC-3′; RPS-F 5′-TACCCTAGCTGAAGGAAAAT-3′; RPL-F 5′- ATGGTTGGAAGATCTAATACCT-3′; RPLa-F 5′-CAACCACTGTCCCCTTCCTA-3′; and RPLb-F 5′-CACCCCAGCAGAGAGAAAAG-3′. LINE-1 primers: 5′-GTCTGGTGTTTGGACCTCCT-3′ (F); 5′-CCGACATGTACGACTCCAGA-3′ (R). Standard PCR reactions for Tsx were cycled for 30–32 cycles. Real-time PCR was performed on a Bio-Rad iCycler maching with SYBR-green iQ Mix (BioRad). Standard curves were generated via amplification of 10-fold plasmid serial dilutions, and ß-actin was used for normalization. Tsix was amplified with primers oNS18 and oNS19 [13] and Xite was amplified with primers NGP3 and NGP4 [12]. Values were normalized to expression of ß-actin.

### Generation of Tsx targeting vector and KO mice

The *Tsx* targeting construct was generated by PCR amplification of three segments of the *Tsx* gene and upstream region, verified by DNA sequencing, then cloned into a modified version of the ploxP-2FRT-PGKneo vector (a gift from Dr. David Gordon via the University of Michigan Transgenic Animal Model Core Facility) that contained a second loxP site between *Kpn*I and *Bam*HI. The upstream *Tsx* fragment (a 4.8 kB section located 6.6 kB upstream of exon 1) was cloned into the *Eco*RI site, the ‘middle’ fragment (1.8 kB upstream of exon 1 and 160 bp of intron 1) was cloned into the *Bam*HI and *Sal*I sites, and the ‘last’ fragment (intron 1 through intron 4 of *Tsx*) was cloned into the *Xho*I site. In summary, a 2.1 kB region encompassing the predicted *Tsx* promoter region upstream of exon 1 and 160 bp of intron 1 was flanked by loxP sites for Cre-mediated deletion of the *Tsx* gene. The neomycin selection marker (under control of the PGK promoter) was flanked by FRT sites, and was used for positive selection (300 µg/mL) of ES cell clones. A novel *Pac*I site was introduced at the end of the last fragment for linearization prior to electroporation. Male TC1 ES cells (derived from 129S6/SvEvTac mice) were electroporated with 20 µg of linearized *Tsx* targeting construct DNA, and 400 neomycin resistant clones were picked. Genomic DNA was isolated from these clones, and digested with *Msc*I for screening via Southern blotting using an external 1 kB probe (Tsx7) overlapping exon 5. Tsx probe 7 was generated using primers Tsx 7 F 5′-GCCTCCACTAGCACATGACA-3′ and Tsx 7 R 5′-CCCTCAGTCCTGCCTCTACC-3′. Three positive clones, containing just one integration of the targeting construct, were obtained, and clone 3A8 was selected for C57BL/6J blastocyst injection at the Brigham & Women's Hospital Transgenic Mouse Facility (Boston, MA). Two chimeric males were obtained following injection, and both transmitted the construct through the germline by matings to C57BL/6J females. Brown female pups were genotyped by Southern blot then mated to male animals expressing FLPe under control of the ROSA26 promoter (129S4/SvJaeSor-Gt(ROSA)26Sortm1(FLP1)Dym/J; Jackson Labs) mice to remove the neomycin selection marker. Animals were genotyped by Southern blot, digesting DNA with *Sph*I and using an internal 1 kB probe (Tsx L2), located between exons 3 and 4. Tsx probe L2 was generated using primers Tsx L2 F 5′-ACATCCCCCATGAAAACTGA-3 and Tsx L2 R 5′-ACCAAAACCAAAACCCAACA-3′. Positive animals were then mated once to C57BL/6J, pups were genotyped using *Sph*I and probe Tsx L2, and positive animals were selected for mating with animals expressing Cre-Recombinase under control of the adenovirus EIIa promoter (B6.FVB-Tg(EIIa-cre)C5379Lmgd/J; Jackson Labs). Following Cre-mediated deletion of *Tsx*, animals were genotyped by Southern blot using *BstZ*17I digestion and probe Tsx L2. Positive animals were then outcrossed to C57BL/6J animals for a total of 6 generations. All animals were weaned at 4 weeks, then tagged and tailed for DNA isolation for genotyping.

### Northern analysis of *Tsx* transcripts

RNA (20 µg/lane) isolated from adult tissues was separated on denaturing 1% agarose-formaldehyde gels for 3 hours at 100V, then transferred to a Hybond-XL membrane overnight. The membrane was pre-hybridized using UltraHyb or buffer (Ambion) for 1 hour at 45°C, followed by overnight hybridization of the ^32^P-radiolabeled probe. The radiolabeled Tsx cDNA probes (both anti-sense and sense orientations consisting of Tsx exons 1–7), and the ß-actin probe were generated by linear amplification using the Ambion Strip-EZ kit and PCR primers Tsx cDNA F2 (sense strand), *Tsx* cDNA R2 5′-ATTGGAAGTTTGGCAAGCAA-3′ (for antisense strand), and ß-actin R. The following morning, the membranes were washed twice with low-stringency buffers (2×SSC/0.1% SDS) followed by two washes with high-stringency buffers (0.1×SSC/0.1% SDS), at 45°C. The membranes were stripped according to the kit instructions then probed for ß-actin. The membranes were visualized by exposing to a phosphoimaging screen. The male testes blot was exposed for 2 hours, and the male lung blot was exposed for 1 day.

### Histology, TUNEL analysis, and immunofluorescence staining

Histological analysis was carried out on 4% paraformaldehyde-fixed testes that were paraffin-embedded, then sectioned at 5 µM thickness. Testis sections were deparaffinized using two changes of Histoclear (National Diagnostics) and hydrated to water by successive 2 min washes in 100% ethanol, 90% ethanol, 80% ethanol, 70% ethanol, and distilled H_2_O. Slides were then incubated in 10 mM sodium citrate pH 6.0 at 100°C for 20 min, followed by 20 min incubation at room temperature. Following unmasking, slides were washed twice in PBS. The TUNEL mixture (In Situ Cell Death Detection kit, Fluorescein; Roche) was incubated for 60 min at 37°C. The slides were washed three times in PBS for 5 min, then mounted with Vectashield mounting media containing DAPI. For immunofluorescence co-staining with SCP1 and Stra-8 antibodies, slides after TUNEL were blocked with 5% BSA in PBS-Tween-20 for 20 min at room temperature. The primary antibody was added (SCP1 at 1∶100 dilution; Stra-8 at 1∶400 dilution) and slides were incubated overnight at 37°C. The next morning the slides were washed three times in PBS-Tween-20 then incubated with a 1∶500 dilution of goat anti-rabbit Cy3 antibody for 30 min at room temperature. Slides were washed three times in PBS-Tween-20 then mounted with Vectashield containing DAPI.

### Tsx KO ES cell derivation and differentiation

Tsx KO male and KO/KO female animals (ages 8–12 weeks) were naturally mated and females were sacrificed at 3.5dpc. Blastocysts were flushed out of the uterine horns and plated on gelatinized 15 cm plates containing mouse embryonic fibroblasts (MEFs) as described in [38]. The inner cell mass (ICM) was dissected five days later, trypsinized in a droplet, and plated onto a fresh well of MEFs. The cells were passaged and expanded until there was sufficient numbers to culture in a T25 flask (a total of 3–4 passages after ICM dissection). The Tsx KO cell lines were genotyped for gender using Zfy, NS18, and NS19 primers as described previously [39]. For differentiation experiments, ES cells were typsizinized and one million cells were plated (in triplicate) in petri dishes in ES medium lacking LIF, using the EB suspension method described previously [8]. Tsx KO cell lines of the same passage number (spanning passage number 6,7,8,9) were used for each differentiation experiment, and four independent differentiations were performed. The medium was changed every two days, and cellular cytotoxicity and viability was determined for 100,000 cells at each time point using the MultiTox-Fluor Multiplex Cytotoxicity Assay (Promega). Cell growth of undifferentiated ES cells was determined by plating 150,000 cells per well (in triplicate) of a 12-well gelatinized plate containing MEFs. The medium was changed daily, and cells were harvested at different time points by trypsinization and counted using a Cellometer (Nexcelom Bioscience).

### Open field task

Spontaneous locomotor activity was monitored using a MED-OFA-MS open field test system (Med Associates, St. Albans, VT). The animal was placed in the center of the activity-field arena, which is a transparent Plexiglas cage (W×D×H; 27×7×20 cm) equipped with three 16 beam infrared transmitter and sensor arrays to register horizontal and vertical activity. Ambient conditions included moderate levels of illumination and white noise (800 lux and 40 dB, respectively). The mouse's position and movement is monitored continuously in the horizontal and vertical planes by dual 16-beam infrared beam arrays. The central zone area was defined as 20×20 cm; the left arena was defined as peripheral zone. Total distance traveled, ambulatory time, ambulatory counts, stereotypy time, stereotypy counts, resting time, vertical counts, vertical time, zone entries, zone time, jump counts, jump time, average velocity, and ambulatory episodes were recorded for each test mouse throughout the 60 min. test session. Total distance provides an index of activity, while the proportion of time or distance spent in the center is taken as a measure of anxiety.

### Elevated plus maze

The elevated plus maze (Med Associates) consists of a plus-shaped runway with two horizontal open arms and two horizontal closed arms (each 6 cm wide×35 cm long) joined by a 6 cm square center platform. The closed arms are enclosed by 20 cm black polypropylene walls. Mice are placed in the center square and allowed to explore freely under ambient light for five minutes. The number of entries and time spent in each arm is recorded. Open arm entries and occupancy provide an inverse measure of anxiety.

### Cued and contextual fear conditioning

The fear conditioning tasks were conducted as described [40], [41]. Training session consisted of a 3 min exploration period followed by three CS-US pairings separated by 1 min (foot-shock intensity 0.8 mA, duration 0.5 s; tone 75 db white noise for 30 sec). Context tests were performed in the same training chamber after retention delays of 1 hr and 24 hr. Tone tests were performed in an environmentally altered testing chamber (different flooring and additional shelter) 24 hrs following training; baseline freezing was monitored (2 min) prior to phasic presentation of the tone (75 db white noise, 3 min duration). Baseline freezing was monitored for 2 min prior to phasic presentation of the tone (75 db white noise, 3 min duration). Mice were trained and tested in conditioning chambers that had a stainless steel grid floor through which footshocks could be delivered (Med Associates, St. Albans, VT). During training and testing sessions, the mouse's position in the chamber is recorded, digitized and analyzed using a video tracking system interfaced with a custom software package. Control and mutant groups consisted of age-matched male and female littermates (8–10 weeks of age) for each analysis. Female animals were group housed (4 animals per cage) for 2 weeks before testing in order to synchronize estrus cycles. Data are presented as group means ± SE. One way and two way ANOVA and Student t-test were used to determine statistically significant differences. For all experiments, the experimenter was blind to genotype.

## Supporting Information

Figure S1 Open field tests and elevated plus maze tests on male *Tsx^KO^* mice. (A) Open field test quantifying the time spent at the periphery (Zone R) of the chamber. (B) Elevated plus maze test quantifying the average number of entries into both open and closed areas. (C) Elevated plus maze test for the average time spent in both open and closed area.
(TIF)

Figure S2 Fear conditioning test in female *Tsx^KO^* mice. Contextual fear conditioning tests for 1 h and 24 h after training. The mean percentage of time spent freezing for one of two independent experiments (yielding similar results) is shown, for both wildtype (n = 9) and Tsx −/− (n = 9) female animals.
(TIF)
