# Supplementary figures and images for: Tsx Produces a Long Noncoding RNA and Has General Functions in the Germline, Stem Cells, and Brain
Source: PLoS Genet. 2011 Sep 1;7(9):e1002248. doi: 10.1371/journal.pgen.1002248 (PMC3164691; doi:10.1371/journal.pgen.1002248)

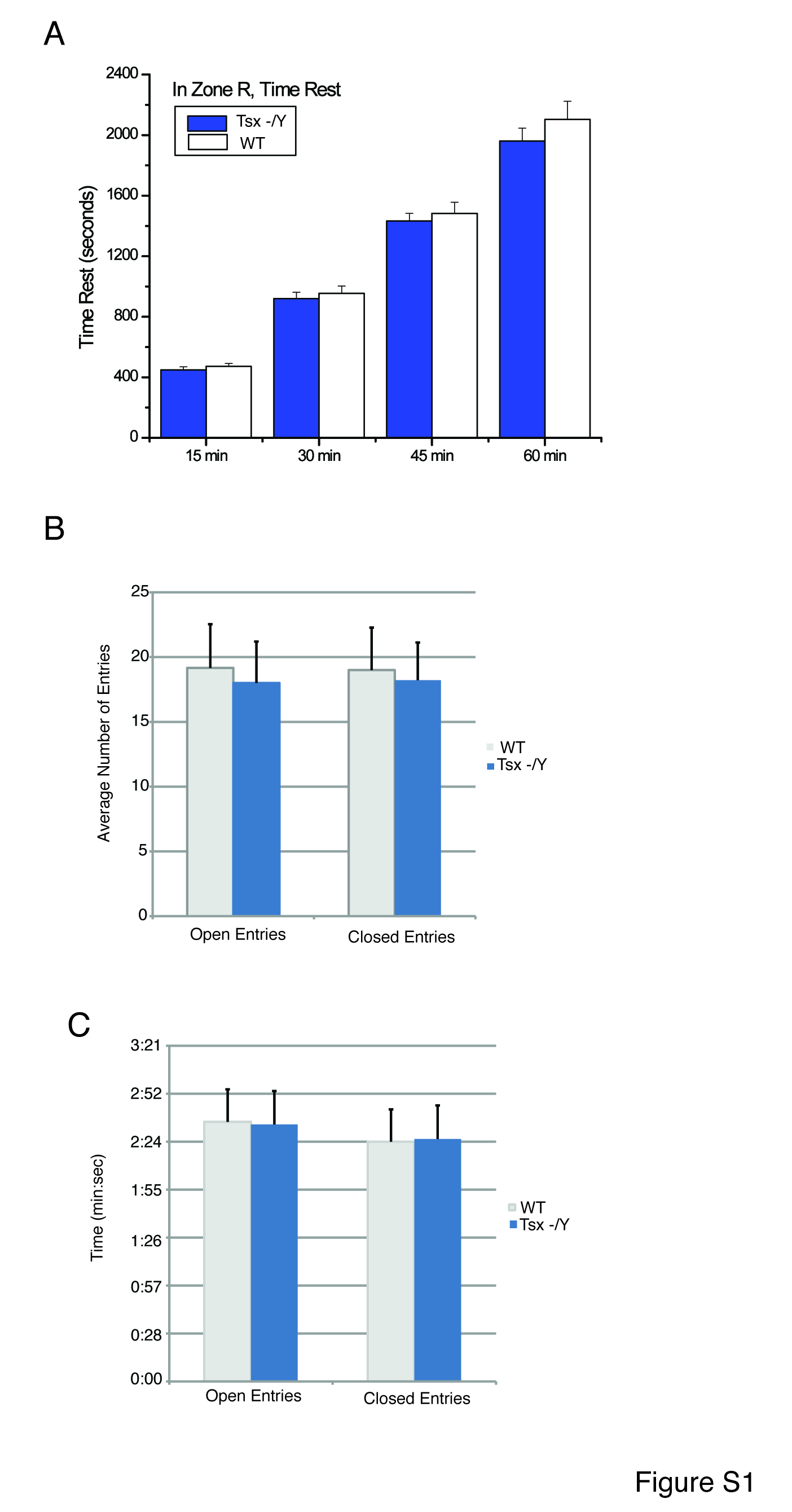

Supplement: Figure S1 — Open field tests and elevated plus maze tests on male TsxKO mice. (A) Open field test quantifying the time spent at the periphery (Zone R) of the chamber. (B) Elevated plus maze test quantifying the average number of entries into both open and closed areas. (C) Elevated plus maze test for the average time spent in both open and closed area. (TIF) [file pgen.1002248.s001.tif]

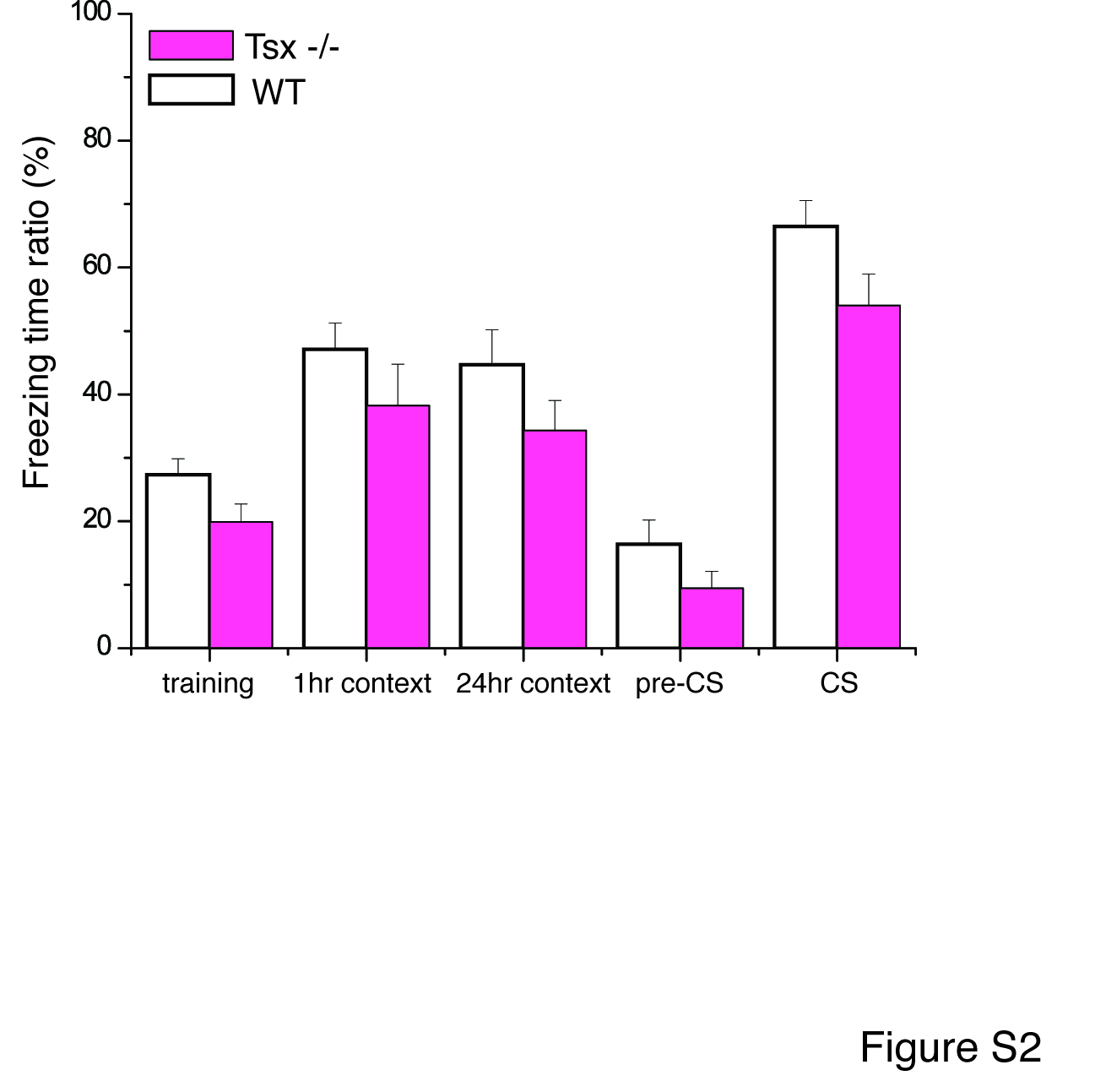

Supplement: Figure S2 — Fear conditioning test in female TsxKO mice. Contextual fear conditioning tests for 1 h and 24 h after training. The mean percentage of time spent freezing for one of two independent experiments (yielding similar results) is shown, for both wildtype (n = 9) and Tsx −/− (n = 9) female animals. (TIF) [file pgen.1002248.s002.tif]
